# (1-Adamant­yl)diphenyl­methano­l

**DOI:** 10.1107/S1600536810021707

**Published:** 2010-06-16

**Authors:** Robert Vícha, Marek Nečas

**Affiliations:** a Department of Chemistry, Faculty of Technology, Tomas Bata University in Zlin, Nám. T. G. Masaryka 275, Zlín 762 72, Czech Republic; b Department of Chemistry, Faculty of Science, Masaryk University in Brno, Kamenice 5, Brno-Bohunice 625 00, Czech Republic

## Abstract

In the title compound, C_23_H_26_O, the adamantane cage consists of three fused cyclo­hexane rings in classical chair conformations with absolute values of the C—C—C angles in the range 106.57 (11)–111.56 (12)°. The dihedral angle between the two phenyl rings is 81.38 (4)°. Although a hy­droxy group is present as a conceivable donor, no hydrogen bonds are observed in the crystal structure.

## Related literature

For the preparation and spectroscopic properties of the title compound, see: Vícha *et al.* (2006 ▶); Stetter & Rauscher (1960 ▶); Molle *et al.* (1984 ▶). For related structures, see: Vaissermann & Lomas (1997 ▶).
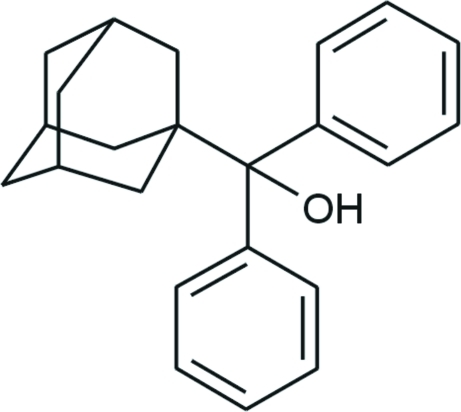


## Experimental

### 

#### Crystal data

C_23_H_26_O
*M* *_r_* = 318.44
Monoclinic, 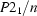

*a* = 6.5370 (12) Å
*b* = 17.037 (3) Å
*c* = 15.322 (2) Å
β = 91.993 (14)°
*V* = 1705.4 (5) Å^3^
*Z* = 4
Mo *K*α radiation
μ = 0.07 mm^−1^
*T* = 120 K
0.40 × 0.40 × 0.30 mm

#### Data collection

Kuma KM-4-CCD diffractometer
Absorption correction: multi-scan (*CrysAlis RED*; Oxford Diffraction, 2009 ▶) *T*_min_ = 0.971, *T*_max_ = 0.978
10354 measured reflections
2996 independent reflections
2133 reflections with *I* > 2σ(*I*)
*R*_int_ = 0.027

#### Refinement

*R*[*F* ^2^ > 2σ(*F* ^2^)] = 0.036
*wR*(*F* ^2^) = 0.095
*S* = 0.93
2996 reflections
221 parameters
H atoms treated by a mixture of independent and constrained refinement
Δρ_max_ = 0.21 e Å^−3^
Δρ_min_ = −0.18 e Å^−3^

### 

Data collection: *CrysAlis CCD* (Oxford Diffraction, 2009 ▶); cell refinement: *CrysAlis RED* (Oxford Diffraction, 2009 ▶); data reduction: *CrysAlis RED*; program(s) used to solve structure: *SHELXS97* (Sheldrick, 2008 ▶); program(s) used to refine structure: *SHELXL97* (Sheldrick, 2008 ▶); molecular graphics: *ORTEP-3* (Farrugia, 1997 ▶) and *Mercury* (Macrae *et al.*, 2008 ▶); software used to prepare material for publication: *SHELXL97*.

## Supplementary Material

Crystal structure: contains datablocks global, I. DOI: 10.1107/S1600536810021707/lh5059sup1.cif

Structure factors: contains datablocks I. DOI: 10.1107/S1600536810021707/lh5059Isup2.hkl

Additional supplementary materials:  crystallographic information; 3D view; checkCIF report

## Figures and Tables

**Fig. 1.**
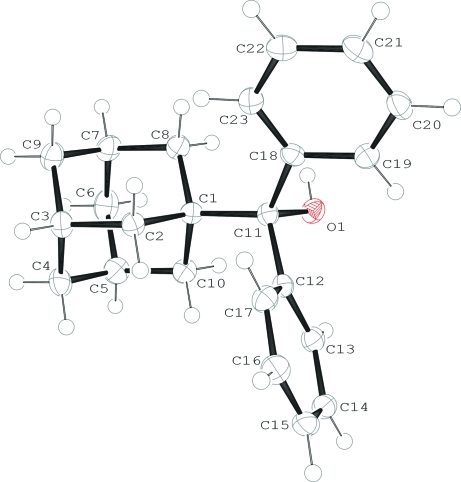
An ellipsoid plot (50% probability) of the asymmetric unit. Hydrogen atoms are represented as arbitrary spheres.

**Fig. 2.**
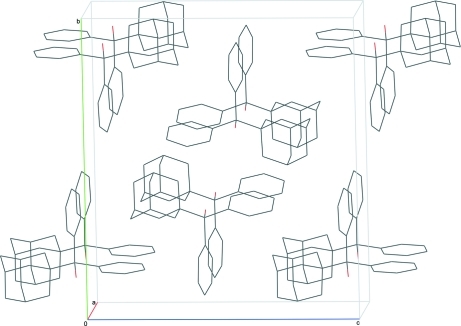
A crystal packing viewed along the a-axis. Hydrogen atoms are omitted for clarity.

## supplementary crystallographic information

### Comment

Adamantane derivatives are well known primarily for their unusual virustatic effect. Nevertheless, the scope of the biological activity of adamantane derivatives is much wider and novel compounds are prepared and tested steadily. The title tertiary alcohol was isolated as unwanted by-product accompanying 1-adamantyl phenyl ketone when no catalyst was employed. The molecule of title compound (Fig. 1) consists of two phenyl rings and an adamantane cage bound to a carbon atom to form a strained tertiary alcohol. Both phenyl rings (C12–C17 and C18–C23) are essentially planar with the maximum deviation from the best planes being 0.0110 (14) Å for C17 and 0.0027 (14) Å for C19. The angle between best planes of these rings is 81.38 (4)°. Both rings are slightly deformed in the plane owing to steric hindrance of the bulky adamantane moiety. The torsion angles describing arrangement of two phenyl rings and the adamantane cage C2—C1—C11—C12 and C1—C11—C12—C13 are -59.06 (14)° and -93.57 (15)°, respectively. Although a hydroxy group is present as a conceivable H-donor, no H-bonds were observed in crystal packing (see Fig. 2). The distance between the closest adjacent O-atoms is 5.2050 (17) Å.

### Experimental

Title compound was isolated from a complex mixture obtained by the reaction of adamantane-1-carbonyl chloride with phenylmagnesium bromide as it has been described previously (Vícha *et al.*, 2006). The crystal used for data collection was grown by slow cooling of a saturated solution of title compound in *n*-hexane.

### Refinement

Carbon bound hydrogen atoms were positioned geometrically and refined as riding using standard SHELXTL (Sheldrick, 2008) constraints, with their U_iso_ values set to 1.2U_eq_ of their parent atoms. The oxygen bound hydrogen atom was located in a difference Fourier map and refined isotropically.

### Crystal data

**Table:**

C_23_H_26_O	*F*(000) = 688
*M**_r_* = 318.44	*D*_x_ = 1.240 Mg m^−^^3^
Monoclinic, *P*2_1_/*n*	Melting point: 400 K
Hall symbol: -P 2yn	Mo *K*α radiation, λ = 0.71073 Å
*a* = 6.5370 (12) Å	Cell parameters from 10305 reflections
*b* = 17.037 (3) Å	θ = 3.1–27.1°
*c* = 15.322 (2) Å	µ = 0.07 mm^−^^1^
β = 91.993 (14)°	*T* = 120 K
*V* = 1705.4 (5) Å^3^	Block, colourless
*Z* = 4	0.40 × 0.40 × 0.30 mm

### Data collection

**Table:**

Kuma KM-4-CCD diffractometer	2996 independent reflections
Radiation source: fine-focus sealed tube	2133 reflections with *I* > 2σ(*I*)
graphite	*R*_int_ = 0.027
Detector resolution: 0.06 mm pixels mm^-1^	θ_max_ = 25.0°, θ_min_ = 3.3°
ω scan	*h* = −7→7
Absorption correction: multi-scan (*CrysAlis RED*; Oxford Diffraction, 2009)	*k* = −20→20
*T*_min_ = 0.971, *T*_max_ = 0.978	*l* = −15→18
10354 measured reflections	

### Refinement

**Table:**

Refinement on *F*^2^	Primary atom site location: structure-invariant direct methods
Least-squares matrix: full	Secondary atom site location: difference Fourier map
*R*[*F*^2^ > 2σ(*F*^2^)] = 0.036	Hydrogen site location: inferred from neighbouring sites
*wR*(*F*^2^) = 0.095	H atoms treated by a mixture of independent and constrained refinement
*S* = 0.93	*w* = 1/[σ^2^(*F*_o_^2^) + (0.0571*P*)^2^] where *P* = (*F*_o_^2^ + 2*F*_c_^2^)/3
2996 reflections	(Δ/σ)_max_ = 0.001
221 parameters	Δρ_max_ = 0.21 e Å^−^^3^
0 restraints	Δρ_min_ = −0.18 e Å^−^^3^

### Special details

**Table:**

Geometry. All e.s.d.'s (except the e.s.d. in the dihedral angle between two l.s. planes) are estimated using the full covariance matrix. The cell e.s.d.'s are taken into account individually in the estimation of e.s.d.'s in distances, angles and torsion angles; correlations between e.s.d.'s in cell parameters are only used when they are defined by crystal symmetry. An approximate (isotropic) treatment of cell e.s.d.'s is used for estimating e.s.d.'s involving l.s. planes.
Refinement. Refinement of *F*^2^ against ALL reflections. The weighted *R*-factor *wR* and goodness of fit *S* are based on *F*^2^, conventional *R*-factors *R* are based on *F*, with *F* set to zero for negative *F*^2^. The threshold expression of *F*^2^ > σ(*F*^2^) is used only for calculating *R*-factors(gt) *etc*. and is not relevant to the choice of reflections for refinement. *R*-factors based on *F*^2^ are statistically about twice as large as those based on *F*, and *R*- factors based on ALL data will be even larger.

### Fractional atomic coordinates and isotropic or equivalent isotropic displacement parameters (Å^2^)

**Table:**

	*x*	*y*	*z*	*U*_iso_*/*U*_eq_	
O1	0.20822 (14)	0.62332 (7)	0.54962 (6)	0.0238 (3)	
C1	−0.0671 (2)	0.63609 (8)	0.65477 (9)	0.0192 (3)	
C2	−0.2658 (2)	0.67842 (8)	0.68106 (9)	0.0215 (3)	
H2A	−0.2439	0.7359	0.6801	0.026*	
H2B	−0.3779	0.6657	0.6383	0.026*	
C3	−0.3275 (2)	0.65309 (8)	0.77314 (9)	0.0249 (3)	
H3	−0.4570	0.6805	0.7881	0.030*	
C4	−0.1578 (2)	0.67522 (9)	0.84012 (10)	0.0297 (4)	
H4A	−0.1979	0.6597	0.8994	0.036*	
H4B	−0.1357	0.7327	0.8396	0.036*	
C5	0.0398 (2)	0.63284 (9)	0.81687 (9)	0.0287 (4)	
H5	0.1516	0.6474	0.8601	0.034*	
C6	0.0060 (2)	0.54372 (9)	0.81919 (10)	0.0326 (4)	
H6A	0.1342	0.5162	0.8053	0.039*	
H6B	−0.0334	0.5275	0.8783	0.039*	
C7	−0.1637 (2)	0.52169 (9)	0.75231 (10)	0.0275 (4)	
H7	−0.1866	0.4637	0.7537	0.033*	
C8	−0.0988 (2)	0.54619 (8)	0.66034 (9)	0.0239 (3)	
H8A	−0.2055	0.5299	0.6166	0.029*	
H8B	0.0301	0.5191	0.6465	0.029*	
C9	−0.3624 (2)	0.56423 (9)	0.77457 (10)	0.0278 (4)	
H9A	−0.4726	0.5500	0.7315	0.033*	
H9B	−0.4054	0.5479	0.8332	0.033*	
C10	0.1005 (2)	0.65811 (9)	0.72486 (9)	0.0241 (3)	
H10A	0.2308	0.6323	0.7105	0.029*	
H10B	0.1228	0.7156	0.7241	0.029*	
C11	0.0093 (2)	0.66125 (8)	0.56147 (9)	0.0195 (3)	
C12	0.0501 (2)	0.75000 (8)	0.55950 (8)	0.0202 (3)	
C13	0.2443 (2)	0.78085 (8)	0.57812 (9)	0.0235 (3)	
H13	0.3561	0.7463	0.5898	0.028*	
C14	0.2764 (2)	0.86159 (9)	0.57982 (9)	0.0270 (4)	
H14	0.4097	0.8817	0.5925	0.032*	
C15	0.1154 (2)	0.91283 (9)	0.56323 (9)	0.0288 (4)	
H15	0.1373	0.9679	0.5655	0.035*	
C16	−0.0776 (2)	0.88315 (9)	0.54334 (10)	0.0292 (4)	
H16	−0.1885	0.9180	0.5315	0.035*	
C17	−0.1100 (2)	0.80241 (8)	0.54063 (9)	0.0247 (3)	
H17	−0.2426	0.7827	0.5258	0.030*	
C18	−0.1246 (2)	0.64101 (8)	0.47894 (9)	0.0211 (3)	
C19	−0.0360 (2)	0.65919 (8)	0.39898 (9)	0.0245 (3)	
H19	0.0992	0.6797	0.3993	0.029*	
C20	−0.1413 (2)	0.64783 (8)	0.31960 (9)	0.0274 (4)	
H20	−0.0782	0.6610	0.2666	0.033*	
C21	−0.3378 (2)	0.61734 (9)	0.31742 (10)	0.0292 (4)	
H21	−0.4101	0.6092	0.2633	0.035*	
C22	−0.4266 (2)	0.59892 (9)	0.39521 (10)	0.0308 (4)	
H22	−0.5614	0.5780	0.3943	0.037*	
C23	−0.3220 (2)	0.61044 (9)	0.47534 (9)	0.0263 (4)	
H23	−0.3865	0.5972	0.5280	0.032*	
H1O	0.191 (3)	0.5765 (11)	0.5483 (11)	0.048 (6)*	

### Atomic displacement parameters (Å^2^)

**Table:**

	*U*^11^	*U*^22^	*U*^33^	*U*^12^	*U*^13^	*U*^23^
O1	0.0194 (5)	0.0248 (7)	0.0275 (6)	0.0000 (4)	0.0027 (4)	0.0009 (5)
C1	0.0199 (7)	0.0181 (8)	0.0197 (8)	0.0010 (6)	−0.0001 (6)	0.0011 (6)
C2	0.0228 (8)	0.0205 (8)	0.0212 (8)	0.0009 (6)	0.0012 (6)	0.0013 (6)
C3	0.0262 (8)	0.0255 (8)	0.0232 (8)	0.0028 (6)	0.0053 (6)	0.0020 (6)
C4	0.0374 (9)	0.0331 (9)	0.0189 (8)	−0.0015 (7)	0.0059 (7)	0.0013 (7)
C5	0.0303 (9)	0.0349 (9)	0.0204 (8)	−0.0023 (7)	−0.0042 (7)	0.0039 (7)
C6	0.0335 (9)	0.0373 (10)	0.0272 (9)	0.0060 (7)	0.0029 (7)	0.0131 (7)
C7	0.0314 (9)	0.0193 (8)	0.0322 (9)	0.0004 (6)	0.0048 (7)	0.0063 (6)
C8	0.0232 (8)	0.0215 (8)	0.0270 (8)	0.0010 (6)	0.0023 (6)	0.0012 (6)
C9	0.0291 (9)	0.0281 (9)	0.0265 (9)	−0.0014 (6)	0.0054 (7)	0.0056 (7)
C10	0.0227 (8)	0.0265 (9)	0.0229 (8)	−0.0006 (6)	−0.0015 (6)	0.0021 (6)
C11	0.0175 (7)	0.0206 (8)	0.0204 (8)	0.0013 (6)	0.0009 (6)	0.0000 (6)
C12	0.0245 (8)	0.0215 (8)	0.0146 (7)	−0.0016 (6)	0.0019 (6)	0.0002 (6)
C13	0.0255 (8)	0.0264 (9)	0.0185 (8)	−0.0014 (6)	−0.0003 (6)	0.0015 (6)
C14	0.0321 (9)	0.0304 (9)	0.0185 (8)	−0.0098 (7)	−0.0004 (6)	−0.0005 (6)
C15	0.0441 (10)	0.0206 (8)	0.0218 (8)	−0.0056 (7)	0.0035 (7)	−0.0001 (6)
C16	0.0370 (9)	0.0233 (9)	0.0274 (9)	0.0032 (7)	0.0023 (7)	0.0042 (6)
C17	0.0253 (8)	0.0244 (9)	0.0244 (8)	−0.0011 (6)	0.0011 (6)	0.0029 (6)
C18	0.0252 (8)	0.0173 (8)	0.0208 (8)	0.0013 (6)	−0.0003 (6)	−0.0018 (6)
C19	0.0273 (8)	0.0216 (8)	0.0247 (8)	−0.0017 (6)	0.0027 (6)	0.0007 (6)
C20	0.0401 (9)	0.0236 (8)	0.0187 (8)	0.0016 (7)	0.0032 (7)	0.0003 (6)
C21	0.0357 (9)	0.0302 (9)	0.0214 (8)	0.0010 (7)	−0.0048 (7)	−0.0038 (7)
C22	0.0283 (9)	0.0337 (9)	0.0300 (9)	−0.0060 (7)	−0.0035 (7)	−0.0048 (7)
C23	0.0261 (8)	0.0297 (9)	0.0231 (8)	−0.0033 (7)	0.0025 (6)	−0.0009 (6)

### Geometric parameters (Å, °)

**Table:**

O1—C11	1.4693 (16)	C9—H9B	0.9900
O1—H1O	0.806 (18)	C10—H10A	0.9900
C1—C8	1.5482 (19)	C10—H10B	0.9900
C1—C2	1.5507 (18)	C11—C12	1.5357 (19)
C1—C10	1.5535 (18)	C11—C18	1.5513 (19)
C1—C11	1.5895 (18)	C12—C13	1.3942 (18)
C2—C3	1.5425 (18)	C12—C17	1.3986 (19)
C2—H2A	0.9900	C13—C14	1.3915 (19)
C2—H2B	0.9900	C13—H13	0.9500
C3—C9	1.531 (2)	C14—C15	1.384 (2)
C3—C4	1.532 (2)	C14—H14	0.9500
C3—H3	1.0000	C15—C16	1.383 (2)
C4—C5	1.532 (2)	C15—H15	0.9500
C4—H4A	0.9900	C16—C17	1.392 (2)
C4—H4B	0.9900	C16—H16	0.9500
C5—C6	1.535 (2)	C17—H17	0.9500
C5—C10	1.5393 (19)	C18—C23	1.3906 (19)
C5—H5	1.0000	C18—C19	1.4079 (19)
C6—C7	1.530 (2)	C19—C20	1.3901 (19)
C6—H6A	0.9900	C19—H19	0.9500
C6—H6B	0.9900	C20—C21	1.385 (2)
C7—C9	1.536 (2)	C20—H20	0.9500
C7—C8	1.5434 (19)	C21—C22	1.380 (2)
C7—H7	1.0000	C21—H21	0.9500
C8—H8A	0.9900	C22—C23	1.399 (2)
C8—H8B	0.9900	C22—H22	0.9500
C9—H9A	0.9900	C23—H23	0.9500
			
C11—O1—H1O	108.3 (13)	C3—C9—H9B	109.7
C8—C1—C2	109.33 (11)	C7—C9—H9B	109.7
C8—C1—C10	107.05 (11)	H9A—C9—H9B	108.2
C2—C1—C10	106.57 (11)	C5—C10—C1	111.56 (12)
C8—C1—C11	111.29 (10)	C5—C10—H10A	109.3
C2—C1—C11	113.60 (11)	C1—C10—H10A	109.3
C10—C1—C11	108.69 (11)	C5—C10—H10B	109.3
C3—C2—C1	110.83 (11)	C1—C10—H10B	109.3
C3—C2—H2A	109.5	H10A—C10—H10B	108.0
C1—C2—H2A	109.5	O1—C11—C12	105.98 (11)
C3—C2—H2B	109.5	O1—C11—C18	106.15 (10)
C1—C2—H2B	109.5	C12—C11—C18	107.24 (10)
H2A—C2—H2B	108.1	O1—C11—C1	107.44 (10)
C9—C3—C4	109.77 (12)	C12—C11—C1	110.09 (10)
C9—C3—C2	109.48 (11)	C18—C11—C1	119.16 (11)
C4—C3—C2	109.72 (12)	C13—C12—C17	118.12 (13)
C9—C3—H3	109.3	C13—C12—C11	121.66 (12)
C4—C3—H3	109.3	C17—C12—C11	120.21 (12)
C2—C3—H3	109.3	C14—C13—C12	120.80 (14)
C3—C4—C5	108.96 (12)	C14—C13—H13	119.6
C3—C4—H4A	109.9	C12—C13—H13	119.6
C5—C4—H4A	109.9	C15—C14—C13	120.47 (14)
C3—C4—H4B	109.9	C15—C14—H14	119.8
C5—C4—H4B	109.9	C13—C14—H14	119.8
H4A—C4—H4B	108.3	C16—C15—C14	119.45 (14)
C4—C5—C6	109.72 (12)	C16—C15—H15	120.3
C4—C5—C10	109.11 (12)	C14—C15—H15	120.3
C6—C5—C10	109.86 (12)	C15—C16—C17	120.31 (14)
C4—C5—H5	109.4	C15—C16—H16	119.8
C6—C5—H5	109.4	C17—C16—H16	119.8
C10—C5—H5	109.4	C16—C17—C12	120.82 (13)
C7—C6—C5	109.20 (12)	C16—C17—H17	119.6
C7—C6—H6A	109.8	C12—C17—H17	119.6
C5—C6—H6A	109.8	C23—C18—C19	117.20 (13)
C7—C6—H6B	109.8	C23—C18—C11	127.72 (13)
C5—C6—H6B	109.8	C19—C18—C11	115.02 (12)
H6A—C6—H6B	108.3	C20—C19—C18	121.63 (14)
C6—C7—C9	109.53 (12)	C20—C19—H19	119.2
C6—C7—C8	109.22 (12)	C18—C19—H19	119.2
C9—C7—C8	109.67 (11)	C21—C20—C19	120.27 (14)
C6—C7—H7	109.5	C21—C20—H20	119.9
C9—C7—H7	109.5	C19—C20—H20	119.9
C8—C7—H7	109.5	C22—C21—C20	118.85 (14)
C7—C8—C1	111.07 (11)	C22—C21—H21	120.6
C7—C8—H8A	109.4	C20—C21—H21	120.6
C1—C8—H8A	109.4	C21—C22—C23	121.22 (14)
C7—C8—H8B	109.4	C21—C22—H22	119.4
C1—C8—H8B	109.4	C23—C22—H22	119.4
H8A—C8—H8B	108.0	C18—C23—C22	120.82 (14)
C3—C9—C7	109.63 (12)	C18—C23—H23	119.6
C3—C9—H9A	109.7	C22—C23—H23	119.6
C7—C9—H9A	109.7		
